# Too noisy, too dark: sleep environment at a typical stroke unit

**DOI:** 10.1093/esj/aakag024

**Published:** 2026-04-13

**Authors:** Noel Schuerch, Claudio L A Bassetti, Marcel Arnold, Albrecht P A Vorster

**Affiliations:** Department of Neurology, Inselspital University Hospital Bern, Bern 3010, Switzerland; Faculty of Medicine, University of Bern, Bern 3008, Switzerland; Department of Neurology, Inselspital University Hospital Bern, Bern 3010, Switzerland; Faculty of Medicine, University of Bern, Bern 3008, Switzerland; Department of Neurology, Inselspital University Hospital Bern, Bern 3010, Switzerland; Department of Neurology, Inselspital University Hospital Bern, Bern 3010, Switzerland

**Keywords:** circadian rhythm, environmental exposure, light, noise, sleep–wake disorders, stroke

## Abstract

**Introduction:**

Sleep–wake disorders are common after stroke and negatively affect stroke outcome. Environmental factors such as noise and light exposure can perturb sleep quality and destabilise circadian rhythm. This study aimed to quantify light and noise exposure of hospitalised patients in a stroke unit and assessed compliance with recommended guidelines.

**Patients and methods:**

Between July and August 2021, noise and light levels were measured in the former stroke unit of the Inselspital Bern. Noise exposure was assessed via the equivalent continuous A-weighted sound pressure level (LAeq), light exposure was assessed using melanopic equivalent daylight illuminance.

**Results:**

World Health Organization recommended noise levels (30 dB at night and 35 dB during the day) were constantly exceeded. Door beds were significantly noisier than window beds during evenings (53 ± 3.3 dB vs 48.6 ± 3.5 dB) and during nighttime (43.4 ± 4.0 dB vs 39.5 ± 4.5 dB). Minimal daytime illuminance of 250 lx melanopic equivalent daylight illuminance was hardly ever reached at door beds (15 ± 30 min) but was reached for about 3 h throughout the day at window beds (186 ± 108 min). The highest quantity of illuminance was reached during late evening hours (18-22 h) for both window and door beds rather than in the mornings (6-10 h).

**Discussion and conclusion:**

These observations suggest that the setting of monitored stroke units is unfavourable for sleep because of insufficient light and excessive noise exposure. Further studies are needed to confirm this observation and assess the associated effects on sleep–wake functions and stroke outcome.

## Graphical Abstract

**Graphical Abstract:**
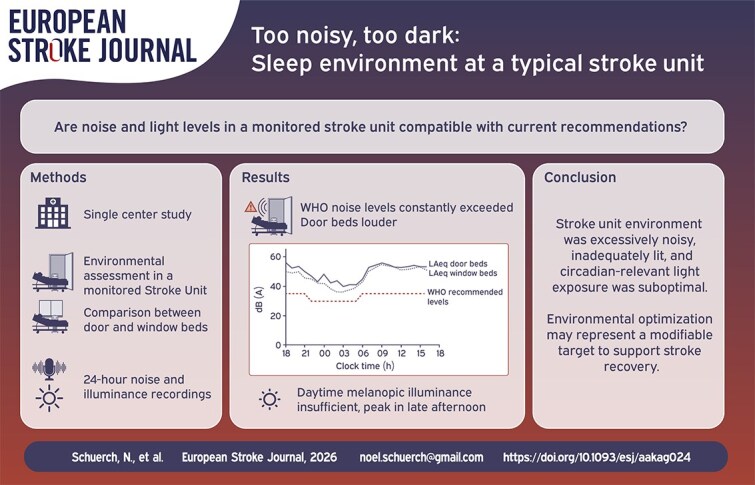

## Introduction

Sleep plays a crucial role in physical, mental and brain health.^1–3^ Sleep–wake circadian functions are modulated by multiple factors such as noise and light.^4,5^

Sleep–wake circadian disorders are highly prevalent in acute stroke patients.^6,7^ Stroke patients have poorer sleep with lower sleep efficiency, shorter total-sleep-time and prolonged wake after sleep onset.^8^ Sleep–wake circadian disorders may not only have a detrimental effect during the acute phase after stroke,^9,10^ they may also have deleterious effects during the subacute and chronic phases after stroke affecting neuroplasticity.^11^ Therefore, high sleep efficiency and low amount of wakefulness after sleep onset are associated with better long-term stroke outcomes.^12^ In particular delirium, a frequent complication in acute phase after stroke linked to prolonged hospitalisation and worse functional recovery, has been associated with sleep disruption.^13^

In the acute phase after stroke patients are best treated in dedicated stroke units led by an interprofessional team.^14,15^ Stroke unit patients receive close neurological, cardiovascular and nursing monitoring, which is essential for optimal care.^16^ However, this high level of surveillance may potentially perturb sleep quality due to increased noise, recurrent awakenings for neurological checks and exposure to artificial light.

The World Health Organization (WHO) has defined thresholds for noise in hospitals. Hospital noise levels should not exceed an equivalent continuous A-weighted sound pressure level (LAeq) of 30 dB at night to not disturb sleep.^17^ However, these levels are likely to be exceeded in hospitals.^18^ In terms of lighting a professional consensus reached at the 2nd International Workshop on Circadian and Neurophysiological Photometry (2019) recommends a melanopic equivalent daylight illuminance (melanopic EDI) maximum of 10 lx during the evening and 1 lx at night and a minimum of 250 lx during the day.^19^ Patients may often be exposed to excessive light during the night and insufficient light during the day.^18^ This may disturb the circadian rhythm of patients and negatively affect mood, cognition and sleep quality.^20^

Major physiological functions such as immune response, haemostasis, cell regulation as well as various biochemical mechanisms follow a circadian rhythm.^21^ A stable circadian rhythm is thought to be crucial for the recovery of stroke patients.^22,23^ It has been shown, that reducing light and noise exposure in intensive care units (ICUs) may significantly reduce delirium and improve sleep efficiency in medical and surgical care patients.^24^

In the absence of studies on these subjects, the aim of this study is to assess the environmental noise and light exposure at a stroke unit with the hypothesis (based on clinical experience) that noise levels may exceed guidelines, and daytime light exposure may be insufficient, while nighttime light exposure may be above recommended levels.

## Patients and methods

**Figure 1:**
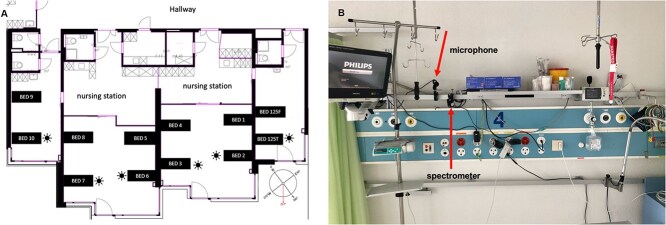
(A) Layout of the former stroke unit at the University Hospital Inselspital Bern. The unit gave room for a total of 12 beds, composed of two 2-bed rooms and two 4-bed rooms. Window beds are marked with a sun symbol; all other beds are classified as door beds. In front of the 4-bed rooms is a nursing station. The windows face a southwest direction (217°, indicated by an arrow). (B) Measurement setup with the microphone and the spectral light detector mounted on a bar behind the bed.

This single-centre observational study was conducted in the former stroke unit of the Neurology Department (two 2-bed rooms and two 4-bed rooms) of the Inselspital, Bern from July 2021 to August 2021 (see Figure 1A for an outline of the stroke unit). In September 2023, the stroke unit has been transferred to a new and modern building and now consists of eight 2-bed rooms. Noise and melanopic illuminance exposure were assessed for all 12 beds in the former unit for at least one 24-h period while the bed was occupied by a patient. A spectral light detector and the microphone were mounted on a bar behind each bed, positioned approximately one metre above the patient’s head (Figure 1B). The sensor was angled at 130° to the vertical plane.

### Noise measurements

Noise was recorded at 1-s intervals using an XL2 audio and acoustic analyser equipped with a M4261 microphone (NTi Audio, Liechtenstein) at each of the 12 beds during weekdays. Each measurement period lasted a minimum of 24 h. Only measurements obtained during bed occupancy were included. Table S1 shows a detailed overview of all measured beds.

The (LAeq) was chosen as the primary noise metric. From the 1-s measurements, hourly LAeq sound pressure levels for each bed were calculated using the Data Explorer Software (NTi-Audio, Liechtenstein).

In addition to LAeq, the hourly A-weighted sound pressure level exceeded for 10% of the measurement period (LA10) and the hourly A-weighted sound pressure level exceeded for 90% of the measurement period (LA90) were calculated based on the LAeq to assess peak and background noise, respectively.

The hourly LAeq, LA10 and LA90 values were further categorised into 3 specific time periods: evening (18:00–22:00), nighttime (22:00–06:00) and daytime (06:00–17:00). Within each period, these hourly measurements were averaged to obtain representative mean sound pressure levels for door and window beds. The interval between 17:00 and 18:00 was excluded from the analysis as this period was used for the daily transition, technical check and read out of measurement devices. If the same bed was measured multiple times, the hourly values were averaged to create a single 24-h dataset per bed. In total, four 24-h datasets were obtained for door beds (from 5 measurement days) and 5 datasets for window beds (from 5 measurement days), each including hourly LAeq, LA10 and LA90 measurements.

### Light measurements

Illuminance, expressed as melanopic EDI, was recorded at 1-min intervals using a CSS-45 spectral light detector (Gigahertz-Optik, Germany) at each of the 12 beds. Measurements lasted at least 24 h and only measurements obtained during bed occupancy were included.

From the 1-min data, half hourly averages were calculated for each bed. For beds that were measured multiple times, half-hourly melanopic EDI values were averaged to create a single 24-h dataset per bed. These datasets were categorised by bed type (door or window beds) to calculate average half-hourly melanopic EDI and representative daytime (06:00–17:00) averages for each category.

**Figure 3:**
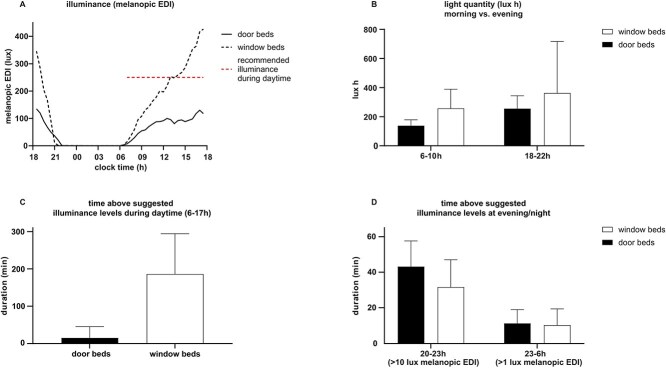
(A) Melanopic equivalent daylight illuminance (melanopic EDI) in lux for window beds (*n* = 5) and door beds (*n* = 4). The dashed horizontal line indicates the minimum suggested melanopic equivalent daylight illuminance during the day of 250 lx. (B) Average summed melanopic equivalent daylight illuminance (melanopic EDI) in lux hours for window beds (*n* = 5, 5 different beds) and door beds (*n* = 4, 4 different beds) for mornings (6–10 h) and evenings (18–22 h). The total light quantity during the night (23–26 h) is not shown. The bars represent group means with the SD. (C) and (D) Total duration (minutes) above suggested illuminance levels for window beds (*n* = 5) and door beds (*n* = 4) during the day 6–17 h (C), during the evening 20–23 h and during the night 23–26 h (D). Window beds reached a longer duration throughout the day (C) than door beds while potentially disruptive illuminance was higher during the evenings and during the night at door beds (D). The bars represent group means with the SD.

The mean peak average half-hourly melanopic illuminance was extracted from the individual datasets and mean peak illuminance was calculated for both bed types to account for timing variability across measurements. The mean half-hourly melanopic EDI curve (Figure 3A) reflects average trends and may not align with individual peak illuminance timings.

In accordance with guidelines proposed by Brown et al.^19^ the duration in minutes for which melanopic EDI exceeded 250 lx (daytime), 10 lx (evening) and 1 lx (nighttime) was calculated for each bed. Each 1-min value was assumed to represent stable melanopic illuminance exposure during that minute. These durations in minutes were also categorised by bed type. Additionally, total light quantities during specific circadian periods (6:00–10:00, 18:00–22:00, 22:00–6:00) were expressed in melanopic EDI lux hours. Time windows of evening and nighttime periods were chosen according to consensus recommendations by Brown et al.^19^ The morning period was set to separately assess the first 4 h of the light period in which light exerts the strongest phase-advancing and alerting effects, as demonstrated by Münch et al.^25^

In total, 9 complete 24-h datasets were included. Four datasets for door beds (from 7 measurement days) and 5 datasets for window beds (from 5 measurement days). Although noise and light were recorded simultaneously at the same bed location, due to incomplete recordings, the final light and noise datasets do not consistently stem from simultaneous recordings.

### Statistical analysis

Hourly sound pressure levels (LAeq, LA10 and LA90) were compared between door and window beds for the 3 defined time periods: evening (18:00–22:00), nighttime (22:00–06:00) and daytime (06:00–17:00). For each time period, all hourly values within the period were included in the analysis (eg, 16 hourly values for door beds and 20 hourly values for window beds during the evening period). Welch’s *t*-test was applied to assess differences between door and window beds, as this method accounts for unequal variances and sampling sizes.

Daytime (06:00–17:00) half-hourly melanopic EDI were compared between door and window beds. For this analysis, all half-hourly values within the period were included in the analysis (eg, 88 half-hourly values for door beds and 110 half-hourly values for window beds during daytime). Welch’s *t*-test was applied to assess differences between door and window beds.

In addition, the durations (in minutes) during which melanopic EDI exceeded the thresholds mentioned above were compared between door and window beds using Welch’s *t*-test. For each threshold, all minute values recorded during the relevant period were included in the analysis.

Data are presented as means ± SD; statistical significance was set at *P* < .05. All statistical tests were performed using Microsoft Excel (Version 16.66.1).

## Results

### Noise

**Figure 2:**
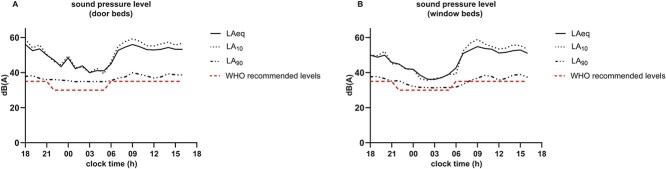
24-h sound pressure level measured in decibels. Average measured hourly sound pressure at (A) door beds (*n* = 4) and (B) window beds (*n* = 5). The dashed line shows the suggested maximum levels by the WHO during the night and day.

Average noise levels, calculated hourly via LAeq at door beds, were consistently high 53.0 ± 3.2 dB(A) during the evening (18:00–22:00), 43.3 ± 4.0 dB(A) at night (22:00–06:00) and 53.0 ± 4.1 dB(A) during the day (06:00–17:00) (Figure 2A). Peak noise levels, measured via the hourly LA10, followed a similar trend 54.8 ± 4.2 dB(A) in the evening, 43.5 ± 4.1 dB(A) at night and 55.7 ± 5.0 dB(A) during the day. In contrast, background noise levels, measured as LA90, were found to be 37.2 ± 2.1 dB(A) in the evening, 35.2 ± 2.2 dB(A) at night and 38.0 ± 2.9 dB(A) during the day. Refer to Table S2 for a comprehensive overview of all noise metrics across the evaluated time periods. LAeq at door beds consistently exceeded the WHO recommended daytime (35 dB(A)) and nighttime (30 dB(A)) limits. Average noise levels (LAeq) did not decrease in the evening, but only at night on average by 10 dB(A).

Window beds exhibited generally lower sound pressure levels than door beds, with an average hourly LAeq of 48.6 ± 3.5 dB(A) during the evening, 39.5 ± 4.5 dB(A) at night and 51.6 ± 4.8 dB(A) during the day (Figure 2B). Evening and nighttime hourly LAeq were significantly lower at window beds compared to door beds (*P* < .001). During daytime hourly LAeq was lower at window beds than at door beds, but the difference was not statistically significant (*P* = .12). The average hourly LA10 values for window beds were recorded at 49.2 ± 5.2 dB(A) in the evening, 39.2 ± 4.6 dB(A) at night and 53.6 ± 6.6 dB(A) during the day. LA10 values at window beds were significantly lower during evenings (*P* < .01) and during the night (*P* < .001) than at door beds but not during daytime (*P* = .08). Background noise levels (LA90) for window beds were 36.8 ± 6.1 dB(A) in the evening, 32.3 ± 2.1 dB(A) during the night, and 36.4 ± 3.8 dB(A) during the day. LA90 values were lower at window beds compared to door beds across all time periods. However, these differences were statistically significant during the day (*P* = .023) and at night (*P* < .001) but not during evenings (*P* = .76).

Despite lower noise levels at window beds, all sound pressure levels still exceeded WHO guidelines.

### Light

Illuminance, measured as melanopic EDI, was consistently higher at window beds (mean 200.9 ± 147.4 lx) compared to door beds (mean 75.0 ± 55.7 lx) throughout the entire day (06:00–17:00; Figure 3A). The difference in daytime melanopic EDI was statistically significant (*P* < .001). The minimum suggested melanopic EDI of 250 lx for window beds was not reached until 13:00, while door beds never achieved this level for any continuous 30-min period (Figure 3A, dashed horizontal line). Peak average melanopic EDI was lower for door beds than for window beds (137.4 ± 74.0 lx vs 479.4 ± 266.1 lx), with peak levels occurring in the late afternoon (16:00–18:30) for both bed types. However, this difference was not statistically significant (*P* = .06). During nighttime hours (23:00–06:00), average melanopic EDI remained below 1 lx for both window beds (0.19 ± 0.34 lx) and door beds (0.18 ± 0.18 lx) with no statistically significant differences observed (*P* = .82). All numerical values are summarised in Table S3.

Window beds achieved the minimum suggested melanopic EDI of 250 lx on average for 186 ± 108 min, compared to just 15 ± 30 min for door beds (06:00–17:00; Figure 3C). Three hours prior to bedtime (20:00–23:00), illuminance exceeded the maximum suggested melanopic EDI of 10 lx for approximately 25% of the time, a total of 43 ± 14.5 min at door beds and for 32 ± 15.4 min at window beds. Interestingly, during nighttime hours (23:00–06:00), recommended maximum melanopic EDI of 1 lx was only slightly exceeded, during 11 ± 7.8 min at door beds and 10 ± 9.2 min at window beds (Figure 3D). Statistically significant was only the difference between door and window beds for daytime (*P* = .021).

The average melanopic EDI lux hours for sensitive circadian time periods (06:00–10:00, 18:00–22:00) are illustrated in Figure 3B. Morning light doses (6:00–10:00) were higher at window beds (258 ± 131.0 lx h) compared to door beds (138 ± 40.9 lx h). Notably, total quantity of illuminance was greater in the evening than in the morning for both bed types (362 ± 353.9 lx h for window beds and 256 ± 88.7 lx h for door beds). During nighttime hours, total light quantity remained low for both bed types (1.5 ± 0.5 lx h for door beds and 1.4 ± 1 lx h for window beds), which is why these data are not depicted in Figure 3D. There was no significant difference in the melanopic EDI lux hours between window and door beds for all time periods (*P* > .05).

## Discussion

To our knowledge, this study is the first to report noise and light levels in a monitored stroke unit. This study reveals that the WHO suggested noise limits for hospitals are constantly exceeded in the stroke unit regardless of the bed location. In terms of lighting, we found that melanopic illuminance levels are insufficient during the daytime, particularly for door beds, while evening melanopic illuminance levels often exceed recommended limits by Brown et al.^19^

Higher sound pressure levels were found at door beds compared to window beds, particularly during nighttime hours. This difference likely reflects the proximity of door beds to the nursing station, suggesting that the primary noise sources are conversations and preparatory activities rather than patient care or medical equipment. These elevated sound pressure levels may lead to sleep disruption and may contribute to the onset of delirium.^26^ Another possible explanation is that critically ill patients are commonly placed closer to the door as they require more frequent monitoring and care. The daytime sound pressure levels found in this study for door beds are comparable to findings for an ICU by Tegnestedt et al.,^27^ while the nightly sound pressure levels found in this study were considerably lower (43.3 ± 4.0 dB(A) vs 52.1–53.4 dB(A)). Yet, this comparison is between a stroke unit and an ICU, which may account for the differences observed.

Even though window beds were more brighter lit during the day than door beds (200.9 ± 147.4 lx vs 75.0 ± 55.7 lx), they rarely met the suggested minimum melanopic equivalent daylight illuminance. Our results are comparable with the illuminance levels found by Leone et al.^18^ Peak melanopic EDI occurred in the late afternoon (16:00–18:30) rather than at noon, with a gradual increase from 07:00 to 16:30 for both bed types. The highest quantity of melanopic equivalent daylight illuminance was seen in the evening rather than in the morning when it would be desirable. For circadian alignment, light dosing during the morning hours should be high and low in the evening hours^28,29^ as light exposure in the evening and at night can lead to unwanted changes in the circadian phase and must therefore be controlled.^30^ Our findings correspond to the southwest-facing orientation (217°) of the stroke unit windows, explaining the greatest melanopic illuminance exposure in the afternoon as the sun sets. In addition, window blinds were frequently kept partially or fully closed during the day, which may have further reduced morning light exposure at window beds.

It is concerning that door beds never reached the required minimum melanopic equivalent daylight illuminance. Inadequate daytime melanopic light exposure can have adverse neuronal effects on cognition, mood and sleep, while increasing melanopic light exposure during the day is beneficial for performance and sleep.^19,20,31^ In addition, door beds exceeded the suggested maximum melanopic illuminance levels in the evening and during nighttime more often than window beds. Therefore, light is suspected to come from the nursing station outside the rooms having a greater impact on door beds compared to window beds. Increased exposure to artificial electrical light during the evening and at night has adverse effects on sleep, circadian rhythms and in general on some health aspects.^32–34^ However, light entering the bedrooms from the nursing station could be easily prevented by consequently closing the blinds. These findings suggest that environmental conditions in stroke units are modifiable factors that may influence circadian regulation and recovery after stroke.

This study has several limitations that should be considered when interpreting the results. No physiological measures (eg, melatonin secretion, cortisol levels or objective sleep assessments) were collected to evaluate the physiological consequences of our observed environmental light and noise exposure. Therefore, the direct circadian impact cannot be determined. Additionally, the study’s timing during the summer, may have led to underestimated sound pressure levels and overestimated melanopic illuminance, as stroke occurrences are typically less frequent during this time of the year.^35^ Thus, an even busier and therefore noisier and darker stroke unit during the winter would be expected.

No patient characteristics for individual beds were collected. However, based on the observations during data collection, noise and light exposure varied predominantly as a function of bed location rather than patient condition. Most noise originated from corridor and nursing-station activity rather than patient-related monitoring.

Furthermore, the placement of the microphone and the spectrometer did not account for patient movement or varying positions in bed. Melanopic EDI was only measured close to the eye level, but not directly at the eye level, like recommended by Brown et al.,^19^ which is necessary for accurately assessing circadian impact. The melanopic illuminance measurement frequency of once per minute may have missed short but potentially high melanopic illuminance exposures, particularly during nighttime. Lastly, the limited sample size, especially for window beds, with only 5 measurements meeting the inclusion criteria, may affect the generalisability of the findings. However, the trends observed suggest that noise and melanopic illuminance exposure in the stroke unit likely present significant challenges to patient recovery.

## Conclusion

These findings reveal that the stroke unit environment was unfavourable for patient recovery. It was excessively noisy and insufficiently lit during the day. Given the critical role that sleep–wake functions play in stroke recovery, the environmental conditions found were not optimal.

For improvement several evidence-based interventions are available including the rigorous closing of patient room doors and the implementation of structured quiet hours. Additional measures may include staff education to raise awareness of noise levels—potentially supported by visual noise metres—as well as the installation of sound-absorbing ceiling and wall materials. Where feasible, spatial separation of nursing stations may further reduce environmental disturbances.

In terms of lighting, we suggest consistent opening of window blinds in the morning and the obligatory use of ceiling lights during the morning hours. Poor lighting can be supported by mountable light boxes or wearable light emitting glasses (eg, Luminette). Although human-centric lighting systems may provide additional benefits, natural daylight from windows remains the strongest and most pleasant light source that is crucial in architectural hospital design.

Future studies should combine environmental measures with physiological circadian markers such as melatonin profiles and core body temperature to directly assess biological impact. Interventional trials have to test whether structured morning light exposure, human-centric lighting systems, or targeted nighttime noise reduction strategies may improve sleep architecture, reduce delirium incidence and ultimately enhance functional recovery.

## Supplementary Material

aakag024_Supplementary_Tables

## Data Availability

The datasets generated during and/or analysed during the current study are available from the corresponding author upon reasonable request.
