# Supplementary material for: Too noisy, too dark: sleep environment at a typical stroke unit
Source: Eur Stroke J. 2026 Apr 13;11(4):aakag024. doi: 10.1093/esj/aakag024 (PMC13131218; doi:10.1093/esj/aakag024)
Supplement: aakag024_Supplementary_Tables [file aakag024_supplementary_tables.pdf]

Supplementary Table S1: Overview of all measured beds.

Bed ID	Bed Type	Noise measurement days	Light measurement days	Included in final noise dataset	Included in final light dataset
B1	Door	2	2	Averaged (1 dataset)	Averaged (1 dataset)
B5	Door	1	1	Yes	Yes
B8	Door	1	2	Yes	Averaged (1 dataset)
B4	Door	1	2	Yes	Averaged (1 dataset)
125 T	Window	1	1	Yes	Yes
B6	Window	1	1	Yes	Yes
B7	Window	1	1	Yes	Yes
B3	Window	1	1	Yes	Yes
B2	Window	1	1	Yes	Yes

Supplementary Table S2: Numerical noise metrics across all time periods

Time period	Sound pressure level (dB)	Door beds	Window beds	*P* value
**evening (18:00-22:00)**	LAeq	53.0 ±3.2	48.6 ±3.5	<0.001
	LA90	37.2 ±2.1	38.8 ±6.1	0.76
	LA10	54.8±4.2	49.2 ±5.2	<0.01
**nighttime (22:00–06:00)**	LAeq	43.3 ±4.0	39.5 ±4.5	<0.01
	LA90	35.2 ±2.2	32.3 ±2.1	<0.001
	LA10	43.5 ±4.1	39.2 ±4.6	<0.001
**daytime (06:00–17:00)**	LAeq	53.0 ±4.1	51.6±4.8	0.12
	LA90	38.0 ±2.9	36.4 ±3.8	0.023
	LA10	55.7 ±5.0	53.6 ±6.6	0.196

Supplementary Table S3: Numerical light metrics across all time periods

Time period	metric	Door beds	Window beds	*P* value
**Morning 06:00-10:00**	Light quantity (lux hours)	138.0 ± 40.9	258.0 ± 13.0	>0.05
**Daytime (06:00-17:00)**	Average melanopic EDI (lux)	75.0 ± 55.7	200.9 ± 147.4	<0.001
	Peak average melanopic EDI (lux)	137.4 ± 74.0	479.4 ± 266.1	0.06
	Time >250 lux (min)	15 ± 30	186 ± 108	0.021
**Evening (18:00-22:00)**	Light quantity (lux hours)	256.0 ± 88.7	362.0 ± 353.9	>0.05
	Time >10 lux (min)	43 ± 14.5	32 ± 15.4	>0.05
	Light quantity (lux hours)	256 ± 88.7	362 ± 353.9	>0.05
**nighttime (23:00–06:00)**	Average melanopic EDI (lux)	0.18 ± 0.18	0.19 ± 0.34	0.82
	Time >1 lux (min)	11 ± 7.8	10 ± 9.2	>0.05
	Light quantity (lux hours)	1.5 ± 0.5	1.4 ± 1.0	>0.05
